# Teachers’ views on the effect of STEM education on the labor market

**DOI:** 10.3389/fpsyg.2023.1184730

**Published:** 2023-06-12

**Authors:** Ahmet Tayfur Akcan, Bekir Yıldırım, Ali Rauf Karataş, Mustafa Yılmaz

**Affiliations:** ^1^Department of International Trade, Faculty of Applied Sciences, Necmettin Erbakan University, Konya, Türkiye; ^2^Department of Mathematics and Science Education, Faculty of Education, Muş Alparslan University, Muş, Türkiye; ^3^Department of Economics, Faculty of Economics and Administrative Sciences, Karabük University, Konya, Türkiye; ^4^Department of International Trade, Faculty of Applied Sciences, Necmettin Erbakan University, Konya, Türkiye

**Keywords:** STEM education, labor market, teacher, workforce, technological unemployment

## Abstract

**Introduction:**

This paper explores teachers’ views on the impact of STEM education on the labor market. This study focused on teachers’ views to investigate STEM education and the labor market and the relationship between the two.

**Methods:**

The sample consisted of 32 teachers from different branches. Participants were recruited using purposive convenience sampling. This paper adopted a qualitative case study research design. Qualitative data were collected using a semi-structured interview form. The qualitative data were analyzed using inductive content and descriptive analysis.

**Results:**

Participants stated that STEM education offered new fields of work, promoted entrepreneurship, and increased job opportunities. They also noted that STEM education helped reduce social costs. They emphasized that STEM education made participants happy, prevented brain drain, and reduced social problems. On the other hand, they also noted that STEM education could lead to technological unemployment. The descriptive analyses showed that STEM education had a positive impact on employment, a reduction in social costs, and a positive impact on underemployment. In light of the results, we made recommendations for future research.

## 1. Introduction

Technological developments and globalization are changing every aspect of life. Technology impacts all areas, from education to the economy and business to social life. These changes lead to the emergence of new education and production models, social lifestyles, and many other areas that are not listed. Globalization and the new world order are changing the labor market and working life. Introducing new production processes into the labor market is a source of economic efficiency and effectiveness. New or updated production processes also push workers to update or renew themselves. Therefore, globalization and innovation bring about differentiation, updating, and openness to innovation in all areas of life. Therefore, In the new world order, employees must be more technically competent than ever. One of the most important ways to achieve this is for employees to receive training appropriate for emerging technical issues and new business processes. In this regard, educational processes should evolve toward more technical subjects early. STEM education helps students develop technical skills at an early age. STEM education is an educational approach that integrates science, technology, engineering, and mathematics by making connections to everyday life (Dare et al., 2019).

STEM education combines scientific and theoretical mathematical knowledge with engineering applications, enabling technological products to emerge (Yıldırım, 2021). New technological products mean new job opportunities. For example, software developers became in high demand because IBM computers were introduced to the market in 1956. Today, we witness the emergence of various professions, such as digital content creators, data analysts, virtual reality creators, etc. (Çepni and Ormancı, 2018). Therefore, new generations should adopt technology. This is realized through STEM education (Sudan, 2021). Therefore, individuals with STEM education will have more job opportunities (Andrée and Hansson, 2020) because it will help students acquire knowledge about different areas and equip themselves with 21st-century skills that will get them jobs in different fields. Other issues that are effective in future planning are globalization and technological advancement. Artificial intelligence automation, machine learning, and robotics are critical issues of the near future. These topics also align with STEM education. Soon, STEM education will cause radical changes in the labor market (Camilli and Hira, 2019). Employees will need to receive specific training in order to adapt to these changes. There are different approaches (design-based learning, creative problem-solving methods, and STEM education) to provide the necessary training for the workforce. Of these approaches, STEM education is one of the best options because it helps students come up with creative solutions and designs to different problems in daily life. Different learning methods, such as problem-based learning and project-based learning are included in the process within the scope of STEM education, which allows students to acquire the knowledge and develop the skills necessary for the business world. STEM education has had an impact on the labor market in a number of different areas (Peters and Jandrić, 2019). STEM education transforms students into more skilled employees who are highly sought after in the labor market (Ring et al., 2017) because it helps them acquire a variety of knowledge and develop the 21st-century skills necessary for the business world. In other words, STEM education increases the number of employment positions for a qualified workforce (Çepni and Ormancı, 2018). Therefore, industries use STEM education to search for and train qualified people for the workforce (Andrée and Hansson, 2020). STEM education prepares the younger generation for challenges (Nguyen et al., 2020). Moreover, STEM education and workforce reform are policies that address labor market failures (Grigorescu et al., 2020). In fact, countries are preparing development plans to address this and increase the number of STEM graduates, because increasing the number of STEM graduates will increase the number of people working in STEM fields.

Research shows that more STEM graduates mean more economic growth and employment (Bacovic et al., 2022). Therefore, people with STEM education are more likely to find jobs. Research even shows that unemployment rates are lower among STEM education graduates (Langdon et al., 2011). People who receive STEM education also develop entrepreneurial aspects and perspectives (Bekki et al., 2018). STEM education positively affects entrepreneurial behaviors (Yaki et al., 2021). In other words, STEM education leads to positive changes for both on the side of employers and on the side of workers.

Moreover, STEM education has much heterogeneity regarding the type and nature of jobs in the labor market. Although each job has different requirements and qualifications, it can be related to STEM education (Salzman and Benderly, 2019, p. 12). This is due to the multidimensional nature of STEM education (Yıldırım et al., 2022). This feature makes STEM education more dynamic, especially regarding the labor market and employment.

### 1.1. Research purpose and significance

Unemployment is a global problem. Especially in developing countries, youth unemployment is high due to a lack of demographic balance (Buheji, 2019). Unemployment will continue to be a macroeconomic problem for nations with varying levels of development, inequitable income distribution, and demographic disparities. Therefore, economic or non-economic policies to reduce unemployment rates will remain important for a long time.

Labor market mobility is reduced by excessive vocational education. People with STEM education do not need job mobility because they are qualified (Yang, 2018). Therefore, unemployment is also prevented by well-designed and planned STEM education programs. Thanks to STEM education, both workers will not be unemployed, and the unemployed will be more likely to find jobs as they will be better able to meet the requirements of vacant job positions. Many researchers emphasize that STEM graduates will have many job options in the future (Çepni and Ormancı, 2018; Yang, 2018; Bacovic et al., 2022). Therefore, from preschool to higher education, STEM education is implemented in formal or informal educational environments (Yıldırım, 2020). Higher education institutions, in particular, are highly involved in STEM education because they profoundly impact regional innovation and development. From various perspectives, higher education institutions represent high investment intensity. Most of this investment is in STEM-related activities (Noonan et al., 2021). More investment means higher labor demand. In doing so, the relationship between STEM education and the working population is also indirectly realized. Therefore, STEM education impacts many areas of the economy, especially the labor market (Yıldırım et al., 2021). A strong link must be established between the STEM education system and economic life for the former to have a greater impact on the latter. However, STEM education remains underrepresented in early education (Allen, 2016). One way of tackling the issue is by teaching children how to question things.

This makes young people more curious and engaged, improving STEM education quality (Decoito and Myszkal, 2018). Early childhood STEM education has many economic benefits. Therefore, this paper focused on teachers’ views on the relationship between STEM education and the labor market. Our results will help us understand how teachers and students perceive STEM education and allow us to make more informed decisions about STEM education content (Arslan, 2021).

In this regard, STEM education has many direct and indirect effects on the labor market (Çepni and Ormancı, 2018). We can better investigate these effects and make more use of STEM education. To this end, the opinions of teachers who have been recipients and providers of STEM education are critical. Few researchers have examined teachers’ views on STEM education and the labor market in detail (Yıldırım et al., 2022). This is what makes this study significant. Therefore, we focused on how teachers view the relationship between STEM education and the labor market. Our results provide answers on further strengthening the link between STEM education and the labor market. Thus, the central question of this study is: “What impact do teachers think STEM education has on the labor market?”

### 1.2. Literature review

#### 1.2.1. STEM education

Today, people must have various skills due to scientific and technological developments. Therefore, countries are integrating different approaches into their education systems, such as creative problem-solving methods, design-based learning, etc. (Arslan, 2021). One of those approaches is STEM education, which supports 21st-century life skills by associating science, technology, engineering, and mathematics with daily life (Sarıoğlu et al., 2022).

Countries are integrating STEM education into their formal and informal education settings for various reasons (Yıldırım, 2021). First, STEM education helps students develop different skills, such as creative thinking, problem-solving, critical thinking, and computational thinking skills. Second, it makes students more interested in science, technology, engineering, and mathematics and encourages them to pursue careers in those fields (Karakaya et al., 2018). Third, STEM education helps students gain the knowledge and skills necessary for future professions (Karakaya and Avgın, 2016). Fourth, STEM education also enables students to come up with new and different products by producing solutions to daily life problems. Therefore, countries need to include STEM education from an early age. Teachers play a key role in the execution of STEM education (Stohlmann et al., 2012). Therefore, we need to provide them with long-term and comprehensive STEM training programs. Some countries have recognized this and attached importance to teacher training.

#### 1.2.2. STEM education and the labor market

STEM education emphasizes the positive aspects of technological progress while ignoring social problems (inequality and injustice) (Roock and Baildon, 2019). Economic growth is also affected by almost all of these positive aspects. STEM education leads to economic growth because it allows us to prosper. STEM education allows fewer employees to do more work because people with interdisciplinary knowledge and skills in STEM education will be able to do more than one job at the same time (Çepni and Ormancı, 2018). This results in lower wage costs. Lower wages mean higher capital investment. High capital investment means new jobs (Zemtsov, 2020). Therefore, through the labor market channel, STEM education has the greatest impact on the economy. We should provide STEM education to students at the earliest possible age to increase this impact. STEM education from an early age will help children develop many characteristics. For example, Earlier STEM education helps students develop the ability to investigate, analyze, solve problems, and explore (Hafizan et al., 2017). These are the very skills that are in demand in the private sector. People are more efficient and effective in their working lives if they develop problem-solving skills early and look at problems from different perspectives. This means that a smaller number of employees can be much more productive. STEM education provides a different perspective on problem-solving and positively impacts planning and success in business. Moreover, students who have received a STEM education are better than others at planning their businesses and carrying out business processes (Ferreira et al., 2021). STEM education instills a sense of entrepreneurship in people of all ages. Shahin et al. (2021) found that STEM education increased the entrepreneurial motivation of female students at the secondary school level. STEM education encourages girls to enter entrepreneurship, especially in developing countries. Therefore, countries should take initiative to encourage women to participate in the workforce (The Girl Scout Research Institute, 2012). STEM education could be a driving force in getting women to participate in the workforce because the labor market has an increasing demand for science, technology, engineering, and mathematics (STEM) disciplines.

STEM education is not meeting this demand to its fullest extent (Almeda and Baker, 2020). Therefore, we need to expand physical spaces for STEM education. However, the larger a classroom is, the less efficient and effective the lessons are. This problem is even more pronounced in STEM fields (Kara et al., 2021). Therefore, schools need to hire more teachers to help make STEM more popular. As economic conditions change, more and more students pursue STEM education. People who receive a STEM education are more likely to be involved in the business world, which increases their employability. On the other hand, there will be an increase in the number of teachers in schools as more and more STEM teachers will be in demand.

Migration also has an impact on the economy and the labor market. STEM education differs from country to country. STEM-oriented schools are the basis of STEM education strategies. This leads people to migrate to cities with STEM-oriented schools (Bullock, 2017). As a result, STEM education also increases internal migration. The relationship between STEM education and the labor market strengthens as migration leads to dynamism. These dynamics have two implications. First, employees and jobs will become more harmonious. Second, everyone will be able to find a job that matches his or her qualifications.

In some countries, employees are overqualified. This means there is a mismatch between theoretical skills and the requirements of business life (Chetwynd et al., 2018). As STEM education becomes widespread, it will generate new job positions, reducing the mismatch between employees and qualifications. This will make employees more productive and help the same number of employees to work more efficiently. In other words, unemployment is likely to increase if students do not receive qualified STEM or similar education because STEM education offers four-way education in an interdisciplinary way: science, engineering, mathematics, and technology.

Students who receive this education develop skills that make it easier for them to get jobs. This reduces unemployment (Widayanti and Suyatna, 2019). In addition, the children of parents who receive a STEM education are more advantaged than the children of parents who do not receive a STEM education (Thomas and Lonobile, 2021). Therefore, both the individuals who receive STEM education and their family members benefit from the prevalence of STEM education. If individuals do not receive qualified education, unemployment among low-educated individuals will remain a problem even with economic growth (Aminu, 2019). In some cases, even STEM education may not be able to eliminate disadvantaged groups (social science majors and women). Moreover, social science graduates have more difficulty entering and staying in the labor market than in other fields (Yang, 2018). After receiving a STEM education, women are less likely to engage in entrepreneurial activities than their male counterparts (Kuschel et al., 2020). These disadvantages show us that we should do a better job of planning STEM education programs. Students can benefit more from STEM education if educators consider these disadvantaged groups in integrating STEM education into curricula. This will further strengthen the link between STEM education and employment. In this way, STEM education can contribute more to the economy.

## 2. Method

This paper focused on both STEM education and the labor market. Addressing topics in all dimensions requires researchers to adopt ontological and epistemological perspectives (Twining et al., 2017). We focused on teachers’ thoughts about how STEM education relates to the workforce. Thus, this study employed both empiricism and interpretation to explain the process. Therefore, we adopted a postpositivist perspective to identify STEM education’s impact on the labor market (Creswell and Poth, 2017). Accordingly, the main research question was: “What are teachers’ views on the impact of STEM education on the labor market?” To answer this question, this study adopted a qualitative single-case research design. In this context, we adopted a single-case research design, which is a qualitative research method, to validate teachers’ opinions about the effects of STEM education on the labor market (Yin, 2014). A case study allows researchers to examine an event in its setting and interpret it holistically (Merriam, 2009). According to Creswell (2007), a case study allows the researcher to examine one or more bounded situations in depth through multi-source data collection tools (observations, interviews, audiovisuals, documents, reports) and to produce qualitative research in which situations and relevant themes are identified. The case of this study is STEM education and workforce. So, In this study, the connection between STEM education and labor force was tried to be explained in line with the views of teachers. Because teachers have a first degree influence on the selection of future professions. When the literature is examined, it is seen that another factor that has an impact on the workforce is STEM education. It is also understood that teachers lack knowledge on how to provide the connection between STEM education and labor force with stem applications. It is important to investigate this situation. For this reason, first of all, teachers were given trainings on stem and workforce, and then the reflections of the workforce and stem trainings given to teachers on teachers’ opinions were determined. We trained teachers for 2 months on STEM education and the workforce. We conducted in-depth interviews with them to identify the relationships between STEM education and the workforce. In other words, we trained teachers to elaborate on the process and collected information on the situation at the end of the process.

### 2.1. Study group

We included teachers who met the predetermined criteria in the study group and sought their opinions. Therefore, we recruited teachers who satisfied the inclusion criteria: (1) having completed the STEM training for 2 months, (2) having participated in all activities related to STEM education and the workforce, and (3) volunteering. The sample consisted of 32 teachers. Participants were teachers from different schools (kindergartens, high schools, etc.). Therefore, they were different branch teachers who taught different subjects. Table 1 shows all participants’ sociodemographic characteristics.

**Table 1:** Sociodemographic characteristics.

Theme	Categories	Codes	*f*
Sociodemographic characteristics	Gender	Woman	24
		Man	8
	Work experience (year)	1–10	14
		11–18	13
		>21	5
	School type	Public	30
		Private	2
	Education (degree)	Bachelor’s	27
		Master’s	5
		PhD	1
	Branch	Science	13
		Math	10
		Classroom	5
		Preschool	2
		Physics	2
	School level	Kindergarten	2
		Primary school	5
		Middle school	17
		High school	8

### 2.2. Data collection tools

#### 2.2.1. Semi-structured interview guide

The data were collected using a semi-structured interview guide developed by the researchers. First, they generated a pool of questions about STEM education and the labor market. The draft consisted of six questions. The researchers consulted two experts to check the intelligibility and relevance of the questions. One of the experts had articles on STEM education, while the other expert had published articles on the labor market. The researchers revised the questions based on expert feedback. They conducted a pilot study with two teachers. They revised and finalized the guide based on their feedback (see Appendix-1: Interview Questions).

### 2.3. Data analysis

Before data collection, the researchers contacted all teachers and informed them about the research purpose and procedure. Then they conducted semi-structured interviews with those who agreed to participate in the study. Each interview lasted 12 to 21 min (544 min in total). The researchers transcribed the interviews. Two experts analyzed the transcripts and developed themes and codes (Yıldırım and Şimşek, 2011). The researchers presented the themes and codes (Tables) in line with the research questions. The researchers ensured intercoder reliability (82%) by getting the two experts to develop the themes and codes (Miles et al., 2014).

The researchers asked all participants to score the questions on a scale of 1 to 5 (according to Table 2) in order to support their views and analyze the interview questions more easily. These questions were asked according to a 5-point Likert type scale, where a maximum score of 5 and a minimum score of 1 can be given, and scaling can be done to determine at what level the participants’ scores will be (Akkuş, 2020). In this way, the researchers checked whether participants’ views were consistent. The researchers interpreted participants’ scores more easily. In this context, the qualitative data were analyzed using descriptive statistics, making it easier for readers to interpret the findings.

**Table 2:** Scores.

	Meaning	Explanation
1–1.8	Strongly disagree	STEM education does not affect the labor market.
1.81–2.6	Disagree	STEM education has no positive effect on the labor market
2.61–3.4	Neither disagree nor agree	Undecided about the effect of STEM education on the labor market.
3.41–4.2	Agree	STEM education has a positive effect on the labor market
4.21–5	Strongly agree	STEM education definitely has a positive effect on the labor market

### 2.4. Validity and reliability

Transferability, credibility, and consistency are critical for validity and reliability (Merriam, 2013). Transferability is achieved by using purposive sampling and explaining the whole research process in detail (Yıldırım and Şimşek, 2011). In the present study, the researchers recruited participants using purposive sampling and explained the whole research process in detail, such as developing the interview guide and collecting and analyzing the data. Experts were involved in the analysis process to achieve credibility. Consistency involves considering findings and conclusions together (Yıldırım and Şimşek, 2011). The researchers provided direct quotes to support their findings in the present study. Moreover, they explained the findings in the Discussion section. All these processes indicate that this study is valid and reliable.

### 2.5. Context

The researchers interviewed all participants after providing them with a 2-month STEM education. Table 3 shows the STEM education process.

**Table 3:** STEM education process.

Week	Topic	Duration
1.	STEM education and its importance	Two hours
2.	Basic concepts related to STEM education	Two hours
3.	STEM pedagogical content knowledge	Eight hours
4.	STEM education and business life	Two hours
5.	STEM education and professions	Two hours
6.	In-class STEM activities	Two hours
7.	Preparing lesson plans	Six hours
8.	STEM education activities	Six hours
9.	Interviews Nine hours four minutes	

## 3. Results

Participants’ answers were presented in Tables. Each table contained direct quotes to help readers interpret the findings (Table 4).

**Table 4:** Participants’ views on the effects of STEM education on the workforce.

Theme	Category	Code	Quotes
The effects of STEM education on the workforce	Positive effects	Promoting the workforce (*n* = 14)	“STEM education has a positive impact on the workforce. People with potential are more sought after and are more likely to be hired by employers.” P1
		Providing the necessary knowledge and skills for working life (*n* = 13)	“STEM education helps people develop problem-solving and entrepreneurial skills. For example, a factory worker might discover a way to use machines more effectively.” P11
		Creating new jobs (*n* = 10)	“Technological developments such as increased automation with artificial intelligence and the Digital Industry create new jobs.” P3
		Creating new professions (*n* = 8)	“Students will face different professions in the future. New professions will emerge soon. For this, I believe that our students should receive STEM education to develop their existing but untapped talents.” P26
		Increasing productivity (*n* = 6)	“STEM education turns students into productive people…” P13
		Contributing to economic growth (*n* = 1)	“STEM education has a significant impact on economic development.” P18
	Negative effects	Causing unemployment (*n* = 2)	“As production increases, labor will not be needed in some areas. But the workforce will increase partly because it creates new job opportunities.” P29
		Technological unemployment (*n* = 1)	“STEM education will help reduce human-related forces because it directly affects technology.” P24

^*^More than one answer.

Participants had different opinions about the impact of STEM education on the workforce. Their views were grouped under two categories: positive and negative. As for positive views, participants stated that STEM education promoted the workforce, provided the necessary knowledge and skills for working life, and created new jobs and professions. As for negative views, they noted that STEM education might leave some people unemployed, which they called technological unemployment. All in all, most participants had positive views regarding the effect of STEM education on the workforce (Table 5).

**Table 5:** Participants’ views on the effects of STEM education on underemployment.

Theme	Category	Code	Quotes
Underemployment	Decreases	Creating new job opportunities (*n* = 17)	“If you have a skilled workforce or if you have 21st-century skills, you know how to survive the pandemic, and you can easily find a job. So, STEM education also reduces underemployment.” P3
		Encouraging people to work (*n* = 11)	“STEM education can prevent underemployment because you can innovate new things and create added value in your sector, field, and discipline. So, it will encourage you to work harder.” P14
		Promoting entrepreneurship (*n* = 7)	“STEM education encourages people to work and bring their products to the market. This helps them develop entrepreneurial skills.” P19
		Having a second job (*n* = 7)	“STEM education reduces underemployment and enables people to have suitable extra jobs.” P20
	Increases	Looking for a new job (*n* = 8)	“People with STEM education are open to interdisciplinary job opportunities because they constantly make connections between different fields. So, they always consider finding new jobs.” P12
		Job dissatisfaction (*n* = 2)	“STEM education helps people develop new skills and be satisfied with their work. This motivates them to work harder.” P24

*More than one answer.

Participants’ views on the effects of STEM education on underemployment were grouped under two categories and six codes. They stated that STEM education created new job opportunities, encouraged people to work, promoted entrepreneurship, and helped people have second jobs. On the other hand, some participants noted that STEM education pushed people to always look for new jobs and caused job dissatisfaction. Most participants believed that STEM education would reduce underemployment (Table 6).

**Table 6:** Participants’ views on the effects of STEM education on discouraged employees.

Theme	Category	Subcategory	Code	Quote
The effects of STEM education on discouraged employees	Positive	Decreases	Creating one’s own job (*n* = 15)	“STEM education allows people to start their own business and helps them develop different skills.” P4
			Creating new jobs (*n* = 12)	“STEM education offers new job opportunities because it addresses multiple disciplines.” P29
			Promoting entrepreneurship (*n* = 9)	“STEM education turns people into entrepreneurs.” P23
			Increasing employment opportunities (*n* = 7)	“STEM education can provide more job opportunities by integrating science, math, technology, and engineering.” P5
			Solving problems (*n* = 5)	STEM education reduces the number of discouraged workers. People with STEM education learn to work hard to solve problems.” P11
			Motivating (*n* = 3)	“Someone who has received STEM education does not fall into such despair because they have improved themselves.” P3
			Skilled workforce (*n* = 2)	“STEM education helps people gain the knowledge and skills needed for the business world. So, they move on to different jobs and keep working.”
	Negative	Partly increases	Promoting entrepreneurship (*n* = 1)	“STEM education increases entrepreneurship partly because it promotes it. People with STEM education try to produce their own products and look for markets for them.” P25
		Increases	Technological unemployment (*n* = 3)	“STEM education increases the number of discouraged workers because market demand is high. I mean, STEM education causes technological unemployment.” P10

*More than one answer.

Participants’ views on the effects of STEM education on discouraged employees were grouped under two categories, three subcategories, and nine codes. Most participants stated that STEM education would reduce the number of discouraged employees as they believed that people who received STEM education would be more likely to start their own businesses because it promoted entrepreneurship and employment. However, some participants believed that STEM education would cause technological unemployment (Table 7).

**Table 7:** Participants’ views of the effects of STEM education on unemployment.

Theme	Code	Quotes
Participants’ views of the effects of STEM education on unemployment	Decreases (*n* = 21)	“STEM education reduces unemployment rates. It reduces the risk of people being unemployed because it is broad …” P2. “Even if people with STEM education become unemployed, it will not last long. They can somehow find jobs or start their own businesses.” P11
	Increases (*n* = 11)	“STEM education may increase the risk of unemployment. Creating new jobs may leave people unemployed.” P13 “STEM education will lead to increased technological unemployment because more and more people will learn how to use technology.” P28

Participants’ views of the effects of STEM education on unemployment were grouped under two codes. Most participants believed that STEM education would reduce unemployment rates. However, some participants remarked that STEM education would cause unemployment because more and more people would learn how to use technology (Table 8).

**Table 8:** Participants’ views of the effects of STEM education on the number of people excluded from the workforce.

Theme	Code	Quotes
The effects of STEM education on the number of people Excluded from the Workforce	Decreases (*n* = 30)	“More people will look for skilled labor. Specialists may be wanted as new jobs will emerge. This means these people specializing in STEM education can be included in the workforce.” P27
	No effect (*n* = 2)	“I do not think it affects that number because STEM education has nothing to do with being excluded from the workforce.” P1

Participants’ views of the effects of STEM Education on the number of people excluded from the workforce were grouped under two codes. Most participants stated that STEM education would reduce the number of people excluded from the workforce. On the other hand, some participants believed that STEM education would not impact the number of people excluded from the workforce (Table 9).

**Table 9:** Participants’ views of the effect of STEM education on social costs.

Theme	Category	Code	Quotes
The effect of STEM education on social costs	Positive	Making one happy (*n* = 20)	“Regarding social costs, people who receive STEM education are more self-confident and morally stronger because they are better equipped. From this point of view, it can also have a reducing effect on social costs.” P26
		Preventing brain drain (*n* = 8)	“STEM education may prevent brain drain and minimize unemployment.” P7
		Alleviating social problems (*n* = 4)	“STEM education will have positive social impacts. The economic cost will be positively affected, and the social cost will be positively affected. This will reduce social problems.” P17
		Reducing suicide rates (*n* = 3)	“People who receive STEM education will have high hopes of finding jobs, which will reduce unemployment rates. Plus, it will reduce suicide rates related to unemployment.”
	Negative	Job dissatisfaction (*n* = 2)	“STEM education helps people acquire new knowledge and skills. So, they want to have good jobs. When they cannot find a job, social costs increase because they will be unhappy.” P19
		No effect (*n* = 1)	“STEM education may not have an impact on social costs. Therefore, I do not think it will have a positive impact.” P7

*More than one answer.

Participants’ views of the effect of STEM education on social costs were grouped under two categories and six codes. Most participants believed that STEM education would help reduce social costs as it would make attendees happier, reduce suicide rates, alleviate social problems, and prevent brain drain. Some participants noted that STEM education would have an adverse impact on social costs (Table 10).

**Table 10:** Descriptive results.

Question No	Question	*M*	SD
1	The impact of STEM education on the workforce	4.47	1.11
2	The impact of STEM education on underemployment	3.81	1.51
3	The impact of STEM education on discouraged workers	4.44	1.34
4	The impact of STEM education on unemployment	3.66	1.62
5	The impact of STEM education on the number of people excluded from the workforce	4.41	0.84
6	The impact of STEM education on social costs	4.22	1.07

### 3.1. Descriptive results

Participants stated that STEM education reduced the number of discouraged workers and those excluded from the workforce. They also noted that STEM education reduced social costs. Moreover, they remarked that STEM education positively impacted underemployment and unemployment. These results indicated that most participants believed that STEM education positively affected the labor market. When we analyze the views of the participants in terms of social cost, it would be appropriate to detail the descriptive results. First of all, STEM education will have a positive effect on social costs. Social costs include an increase in crime rates, dissolution in the social structure, unbalanced migration flows, and suicide. According to our participants, STEM education will positively affect social costs as people will have more job opportunities. Similarly, individuals who develop themselves through STEM education will not be discouraged workers as they will be able to find jobs thanks to their knowledge and skills. Moreover, individuals with improved knowledge and skills will reduce the number of those who cannot enter the labor force.

## 4. Discussion and conclusion

This section discussed the results regarding the impact of STEM education on the labor market.

According to the first result in line with the main research question, most participants believed that STEM education affected the labor market positively. They noted that STEM education promoted the workforce, created new jobs and professions, and helped students acquire the necessary skills and knowledge for the business world. Research also shows that STEM education creates new jobs and professions and helps students acquire the necessary skills and knowledge for the business world (Camilli and Hira, 2019; Andrée and Hansson, 2020). Bacovic et al. (2022) reported a positive correlation between the increase in STEM graduates and economic growth and employment. Talwar and Hancock (2010) emphasized that different professions, such as space pilots, will be popular in the future. However, some participants believed that STEM education might affect the labor market adversely. They remarked that STEM education would cause technological unemployment. Çepni and Ormancı (2018) argue that technological unemployment may occur due to the disappearance of some business areas with technological advances. Yıldırım et al. (2021) also maintain that STEM education will accelerate technological developments, reducing human-based jobs and increasing unemployment. Our results are consistent with the literature.

According to the second result in line with the main research question, participants stated that STEM education would reduce underemployment as they believed that it would create new jobs, promote entrepreneurship, encourage people to work hard, and get them to find second jobs. However, some participants noted that STEM education would increase underemployment rates as they believed it would cause them to be unhappy with their jobs and seek new job opportunities. Bekki et al. (2018) emphasized that STEM education helped students develop entrepreneurial skills and changed their views about finding jobs. Yaki et al. (2021) also maintain that STEM education encourages students to develop entrepreneurial skills. Our results are consistent with the literature.

According to the third result in line with the main research question, most participants noted that STEM education reduced the number of discouraged workers. They stated that STEM education created new jobs, promoted entrepreneurship, increased employment, and encouraged people to solve problems and start their own businesses. Yang (2018) remarked that people who received STEM education did not need job mobility as that education equipped them with the necessary skills. Moreover, Bekki et al. (2018) highlighted that STEM education provided people with entrepreneurial skills necessary for the business world. On the other hand, some of our participants believed that STEM education partially affected discouraged workers or increased the number of discouraged workers. Our results are consistent with the literature (Yaki et al., 2021; Barau et al., 2022).

According to the fourth result in line with the main research question, most participants remarked that STEM education reduced the number of unemployed people. Research shows that STEM education provides new lines of business (Kertil and Gurel, 2016; Widayanti and Suyatna, 2019; Yıldırım et al., 2021). Fahmy and Naqvi (2022) state that there will be more STEM-related lines of business in the future. However, some participants believed that STEM education would cause technological unemployment. Çepni and Ormancı (2018) also argue that technological advances thanks to STEM education will cause technological unemployment. Although some researchers report a positive correlation between STEM education and technological unemployment, many others maintain that STEM education will result in more job opportunities, reducing unemployment rates (Talwar and Hancock, 2010; White and Shakibnia, 2019; Mystakidis and Christopoulos, 2022). Our results are consistent with the literature.

According to the fifth result in line with the main research question, most participants stated that STEM education would reduce the number of those not included in the labor force. However, some participants did not believe that STEM education would affect people excluded from the workforce. Fahmy and Naqvi (2022) maintain that STEM education will reduce the number of people not in the labor force. Langdon et al. (2011) also argue that STEM education graduates are less likely to experience unemployment. Our results are consistent with the literature.

According to the sixth result in line with the main research question, participants stated that STEM education affected social costs positively. Participants noted that STEM education made people happy, prevented brain drain, and reduced social problems and suicide rates. This is because STEM education helps people improve themselves and makes them more equipped to find jobs that fit their skills. However, some participants believed that STEM education would make people dissatisfied with their jobs because they would be overqualified. Some participants noted that STEM education would not affect social costs. Our results are consistent with the literature.

The seventh result in line with the main research question showed that STEM education would positively impact the workforce and discouraged workers and those excluded from the workforce. The results suggested that STEM education would reduce social costs and the number of those excluded from the workforce. Moreover, the results showed that STEM education would reduce underemployment and unemployment rates. Participants’ views and their descriptive analysis results are consistent.

## 5. Limitations and recommendations for further research

This study had three limitations. First, the results are sample-specific and cannot be generalized to the whole population. Second, the results are based on self-report. Researchers should adopt mixed-method research designs to better understand what teachers think about the impact of STEM education on the labor market. Third, we focused only on the impact of STEM education on the labor market. Researchers should conduct further research to better understand the impact of STEM education on different types of unemployment (e.g., technological unemployment) and new professions.

## 6. Implications for future research on STEM education

Some implications for future research were made in light of the results.

1. We need to focus on STEM training because teachers are responsible for delivering STEM education. In this way, we can make STEM education more popular and widespread.
2. We should use materials, 3D printers, and robotic coding materials that will attract students’ interest in STEM fields and enable them to produce solutions to real-world problems.
3. We should encourage students to attend seminars about career opportunities and future professions in STEM fields. We should also organize seminars that address the link between STEM professions and STEM education. Such seminars can help students to plan their careers in STEM fields.
4. Schools should allocate more budget for STEM education and provide environments where teachers can work collaboratively.

## Data availability statement

The original contributions presented in the study are included in the article/supplementary material, further inquiries can be directed to the corresponding author.

## Author contributions

All authors listed have made a substantial, direct, and intellectual contribution to the work and approved it for publication.

## Conflict of interest

The authors declare that the research was conducted in the absence of any commercial or financial relationships that could be construed as a potential conflict of interest.

## Publisher’s note

All claims expressed in this article are solely those of the authors and do not necessarily represent those of their affiliated organizations, or those of the publisher, the editors and the reviewers. Any product that may be evaluated in this article, or claim that may be made by its manufacturer, is not guaranteed or endorsed by the publisher.

## 7. Appendix-1: Interview questions

1. How do you think STEM education affects the workforce?
2. How do you think STEM education affects underemployment?
3. How do you think STEM education affects discouraged workers?
4. How do you think STEM education affects unemployment?
5. How do you think STEM education affects the number of unemployed people?
6. How do you think STEM education affects social costs?
